# Acoustic assessment of erygmophonic speech of Moroccan laryngectomized patients

**DOI:** 10.11604/pamj.2015.21.270.4301

**Published:** 2015-08-11

**Authors:** Naouar Ouattassi, Najib Benmansour, Mohammed Ridal, Zouheir Zaki, Karima Bendahhou, Chakib Nejjari, Abdeljabbar Cherkaoui, Mohammed Nouredine El Amine El Alami

**Affiliations:** 1 ENT Head and Neck Department, Hassan II University Hospital, Fez, Morocco; 2 Epidemiology, Clinical Research and Community Health Department, Faculty of Medicine and Pharmacy, Fez, Morocco; 3 Automatic, Signals and Systems Department, ENSA Tangers, Morocco

**Keywords:** Erygmophonic speech, perturbation analysis method, formants, spectrogram

## Abstract

**Introduction:**

Acoustic evaluation of alaryngeal voices is among the most prominent issues in speech analysis field. In fact, many methods have been developed to date to substitute the classic perceptual evaluation. The Aim of this study is to present our experience in erygmophonic speech objective assessment and to discuss the most widely used methods of acoustic speech appraisal. through a prospective case-control study we have measured acoustic parameters of speech quality during one year of erygmophonic rehabilitation therapy of Moroccan laryngectomized patients.

**Methods:**

We have assessed acoustic parameters of erygmophonic speech samples of eleven laryngectomized patients through the speech rehabilitation therapy. Acoustic parameters were obtained by perturbation analysis method and linear predictive coding algorithms also through the broadband spectrogram.

**Results:**

Using perturbation analysis methods, we have found erygmophonic voice to be significantly poorer than normal speech and it exhibits higher formant frequency values. However, erygmophonic voice shows also higher and extremely variable Error values that were greater than the acceptable level. And thus, live a doubt on the reliability of those analytic methods results.

**Conclusion:**

Acoustic parameters for objective evaluation of alaryngeal voices should allow a reliable representation of the perceptual evaluation of the quality of speech. This requirement has not been fulfilled by the common methods used so far. Therefore, acoustical assessment of erygmophonic speech needs more investigations.

## Introduction

Total laryngectomy performed for advanced stages of laryngeal or hypopharyngeal cancer affects drastically both respiration and phonation. Therefore, voice rehabilitation is among the most prominent contributions to the quality of life. In fact, through history many rehabilitative techniques have been set up but only three of them remained. Erygmophonic speech, tracheoesophageal speech and electolarynx are the most commonly used alaryngeal voicing types nowadays. Both erygmophonic and tracheoesophageal voices show more aperiodicity than normal laryngeal speech. Also, the vocal characteristics of alaryngeal voices are notoriously difficult to extract. In both tracheoesophageal and erygmophonic voices the sound source remains the pseudoglottis. Tracheoesophageal speech is known to have better perceptual qualities, louder and longer phonation with no need for voice training as compared to erygmophonic speech. Nevertheless, the latest remains of great interest to many researchers. It's known that the erygmophonic voice demonstrates perceptual qualities of hoarseness, short phonation duration, and low pitch and volume [1], all of which are indicators of aperiodicity in the voice signal. Perturbation analysis of this perceptual abnormality has been accomplished, with results confirming the severe irregularity of erygmophonic voices [1–3]. However, rehabilitated erygmophonic speech exhibits acceptable to good perceptual qualities. In fact, some patient's performance is quite impressive since they can produce comfortably a perfectly intelligible speech. These patient's are called “excellent oesophageal speakers” [4]. The aim of this paper is to assess the quality of rehabilitated erygmophonic voice of Moroccan speakers through speech training process. Voice quality appraisal was performed by acoustical parameters using perturbation measures method and formantic characteristics using linear predictive coding algorithms.

## Methods

We have conducted a prospective case- control study during a year that investigated the acoustical features of erygmophonic speech of Moroccan laryngectomized patients under rehabilitation speech training at speech therapy unit of ENT Head and Neck Surgery Department, Hassan II University Hospital of Fez. Only patients that joined the rehabilitation protocol were included.

**Subjects:** we included eleven laryngectomized patients that underwent total laryngectomy and adjuvant radiotherapy for locally advanced laryngeal cancer. A group of age matched laryngeal speakers, healthy volunteers with no history of voice, hearing or speech problem, were included as a control group. The two groups’ mother tongue was Arabic and they also have a good master of French. They were all adult male ranging in age from 45 to 65 years old (mean 52.8). All participants approved and consent the procedure used in this study.

**Procedure:** audio samples of the eleven laryngectomized patients were collected each three months since the beginning of the speech training. At each recording session, both normal and erygmophonic speakers were instructed to read a text in Arabic, another in French and to sustain the phonation of the vowel /a/ at a comfortable pitch and volume for as long as possible. Each vocal performance was recorded 3 times. Only the best performance was retained for acoustical analysis. Recording sessions were made in a quiet room with a digital voice recorder (IC Recorder, ICD-PX720) positioned 10cm from the person's mouth. To minimize the recording of stoma noise for erygmophonic speakers, the recorder was placed at an angle of 30°. Audio files were recorded at a sampling rate of fs= 44.1 KHz using the Digital Voice Editor of the same device. For analysis we have excluded the onset and offset of phonation to avoid effects of speech intonations. We applied perturbation measures to normal and erygmophonic voices and assessed formant values through linear predictive coding.

**Acoustic analysis:** perturbation analysis was performed on MATLAB software version 7.0. We proceeded to the analysis of perturbations with the assessment of jitter and shimmer. Jitter measures the cycle-to-cycle frequency variation of a voice signal, while shimmer measures the cycle-to-cycle amplitude variation. Also we obtained the first four formant values (F1, F2, F3 and F4) for vowels using linear predictive coding algorithm.

**Statistical analysis:** Epi info 3.4 software was used for statistical analysis of acoustical data of erygmophonic and normal voice samples. Non-parametric Mann-Whitney rank sum tests were used to compare acoustical parameters of erygmophonic and normal voices. Statistical significance was set at the level of 5% for all tests.

## Results

**Figure 1:**
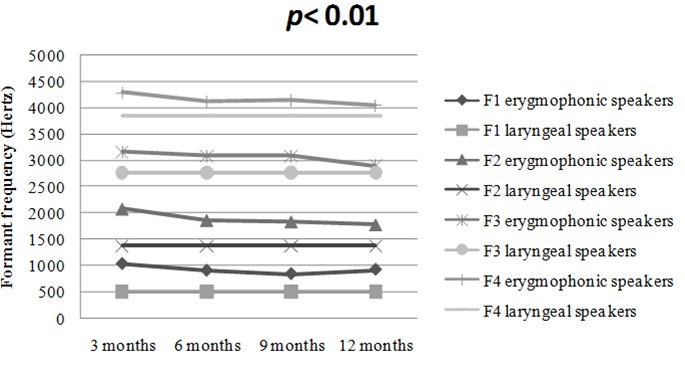
Average formant values of sustained /a/ produced by Moroccan erygmophonic and laryngeal speakers; (p: statistically significant at 0.05 level)

Average values of fundamental frequency (F0), first, second, third and fourth (F1, F2, F3 and F4) formant frequency values of the sustained vowel /a/ produced by Moroccan erygmophonic speakers are reported in Table 1. For visual comparison, the average values and the general trend of sustained /a/ formant values of erygmophonic versus laryngeal speakers are reported in Figure 1. On the basis on these results we have concluded that erygmophonic speakers exhibited higher formant values compared to age matched laryngeal speakers. Also, there was a statistically significant enhancement of anterior formants (F3, F4) versus posterior ones (F1, F2) through rehabilitation course. Furthermore, in comparison to the normal voice, the esophageal voice produces an aperiodic waveform. Therefore, pitch extraction is difficult. Results of perturbation analysis of normal and erygmophonic voices are summarized in Table 2. Erygmophonic voice exhibits higher and extremely variable Error values (mean= 79.3, Standard Deviation= 53.1), and all err values for erygmophonic voice were greater than the acceptable value of 10. Because err indicates the reliability of results, values of mean jitter and shimmer were considered to be highly questionable. However, Err count for perturbation measures of all normal voice samples was 0, indicating that perturbation measures were reliably calculated for these nearly periodic signals. Nonparametric Mann-Whitney rank sum tests on perturbation data indicated significant differences between normal and erygmophonic voices (pFigure 2, Figure 3, respectively. Although the normal voice exhibits well-defined onset of voicing and formant structure that are presented as dark horizontal bands, the erygmophonic voice spectrogram shows a disordered structure, with low extent and poorly defined onset of voicing compared to the normal voice. However, the formant structure is respected with highest formant values compared to laryngeal voice.

**Figure 2:**
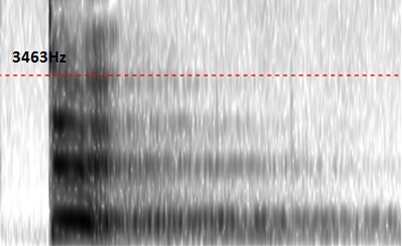
The spectrogram of the laryngeal production of “te” exhibits a well-defined onset of voicing and a formantic organisation

**Figure 3:**
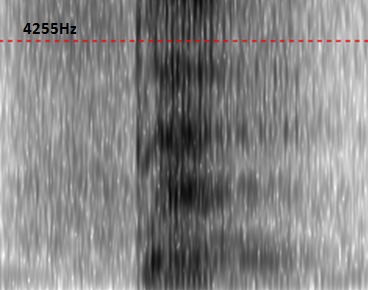
The spectrogram of “te” for a patient at the end of the speech training shows higher F4 frequency, low extent of voicing and observance of the formantic organization

**Table 1:** Average values of fundamental frequency (F0), first, second, third and fourth (F1, F2, F3 and F4) formant frequency values of the sustained vowel /a/ produced by Moroccan erygmophonic speakers

	F0	F1	F2	F3	F4
3 months	285 (17)	1024 (45)	2081 (48)	3159 (68)	4295 (36)
6 months	204 (23)	898 (128)	1858 (134)	3079(142)	4125 (78)
9 months	257 (65)	834 (143)	1830 (127)	3090(183)	4147 (112)
12 months	239 (75)	*916 (215)*	1773 (213)	2903(227)	4055 (245)
	***p*** **: 0.7**	***p: 0.054***	***p*** **: 0.052**	***p*** **: 0.05**	***p*** **: 0.025**

***p*** is the statistical significance of the enhancement of formants values through rehabilitation process.

**Table 2:** Results of perturbation analysis of normal and erygmophonic voices

	Laryngeal voice	Erygmophonic voice *(4th recording at 24 months of rehabilitation course)*	Mann-Whitney Rank Sum Test Results (p*)
Mean Jitter for Arabic (Hz)	M= 112	M= 0.95	N= 33
	SD= 2.75	SD= 0.36	*p*<0.001
Mean jitter for French (Hz)	M= 91	M= 0.593	N= 33
	SD= 5.87	SD= 0.51	*p*<0.001
Mean shimmer for Arabic (dB)	M= 0.75	M= 0.324	N= 33
	SD= 0.3	SD= 0.21	*p*= 0.04
Mean shimmer for French (dB)	M= 0.81	M= 0.297	N= 33
	SD= 0.21	SD= 0.33	*p*= 0.01
Err count	M= 0	M= 79.3	N= 33
	SD= 0	SD= 53.1	*p < 0.001*

Abbreviations: ***M*** **:** Mean; ***SD***: standard deviation; ***N***: the amount of speech samples; ***Err*** **:** error. (*): statistical significance set at the level of ***p*** **=0.05**

## Discussion

Although tracheoesophageal puncture is the preferred method of voice restoration post laryngectomy in the United States and Europe [5], erygmophonic voice has upsurged lately. In fact, it remains a significant laryngectomee voice production method in Asian countries, such as China [6]. Jacobson et al noted the difficulties of trachea-oesophageal speech attainment in non-English/French speakers [7]. It is possible that language-related issues may result in the usage of alternative methods of voice restoration in China. Although Arabic isn't a tone language as Chinese languages (Mandarin, Thai and Cantonese), it still phonetically different from English or French as many phonemes (especially velo-pharyngal ones) such as (*hã*), (*qãf*), (*‘ayn*) don't exist in western languages. It seems to us that erygmophonic performance for these phonemes is perceptually better than tracheoesphageal speech. Currently, in medical care, interest is focused on the objective characterization of alaryngeal voices. The main goal is to provide a reliable alternative of perceptual evaluation of voice quality based on acoustical parameters. However, to the present, voice perceptual evaluation remains the essential way to assess the suitability of the objective appraisal of the quality of speech. In fact, the performance of acoustic parameters is examined through its mathematical correlation to the perceptual evaluation. As objective assessment of erygmophonic speech, three methods have been used so far. The perturbation analysis methods that measure jitter and shimmer's values, the formant characterization of vowels production through linear predictive coding and the representation of the temporal evolution of the broadband spectrum of the speech signal called spectrogram [8–11]. Using perturbation analysis methods, studies have found erygmophonic voice to be significantly poorer than alternative forms of alaryngeal speech and normal speech [6]. Nevertheless, erygmophonic speech is alaryngeal, it does not involve true vocal fold vibrations. It is the controlled burping up of air from the esophagus to produce voice via the vibration of the pharyngo-esophageal segment. This results in imprecise and slow pharyngo-esophageal segment movement [12] and causes significant acoustic differences from normal vocal fold vibration. This suggests a certain degree of aperiodicity which leaves a doubt on the reliability of perturbation analysis methods results. In fact, it has been determined that values of jitter and shimmer can't reliably evaluate aperiodic voice samples [6, 13]. Also, studies using perturbation measures for analysis of extremely aperiodic voices such as erygmophonic speech rarely quantities the reliability of results [13].

A measure of perturbation analysis reliability is the “err count” that was quantified by the Epi info 3.4 software, an indicator of either inaccurate pitch estimation or highly aperiodic signal waveform. All laryngeal voice samples exhibited “err counts” values of 0, which shows low error in analysis. On the other hand, “error counts” for erygmophonic speech samples were high suggesting the lack of reliability of perturbation analysis indicators and supporting the assertion that perturbation analysis methods must be applied with caution to aperiodic signals such as erygmophonic speech. Erygmophonic speech is known to exhibit higher values of formant frequencies. This has been found in several languages such as Mandarin, Spanish, English and Dutch [14]. That has been also confirmed in Arabic. According to the source-filter theory, the length of the vocal tract can significantly affect the formant characteristics of vowels. The shorter the vocal tract length is, the higher the formant frequencies. Therefore, the higher formant frequencies in esophageal speech are believed to be due to a shortened effective vocal tract length in erygmophonic speakers compared to laryngeal speakers [15, 16]. Formant structure is respected in erygmophonic speech, it might seem an accurate method for the assessment of erygmophonic voice quality since pitch structure and formant values are related to perceptual quality of speech. However, formant frequency characterization of vowels might not be the accurate way to assess the quality of erygmophonic speech since speech is more complex than a vowel produced in a given pitch and volume. In fact, acoustic parameters of vocal dysperiodicity are often extracted from stationary fragments of sustained vowels. The reason is that the vowels without attacks and declines are easy to analyze because the assumptions of stationarity and cyclicality used by analytical methods are valid for many authors [17]. Thus we assume that sustained vowels are produced holding time-invariant the characteristics of the voice source, the vocal tract and the articulators. Hence, the parameters of disturbance and noise are easily calculated. Therefore, the use of sustained vowels is popular because of technical feasibility and not due to clinical relevance. However, using sustained vowels to assess the quality of the speech has many drawbacks. First, several methods require a long lasting vocal samples which make them susceptible to variance of pitch and volume. Thus, the assumption of stationarity might be not respected and then reliability of analytical methods results might be questioned. On the other hand, clinicians believe connected speech to be more informative about the interaction between the vocal source and the vocal tract that behaves as resonator. Therefore, connected speech shows the dynamic features of the vocal source and the vocal tract such as attacks and declines and variation of pitch and amplitude. Moreover, speech perceptual assessment is performed upon connected speech. Another way to approach the quality of the voice is the spectrogram. In fact, a spectrogram is a display of the frequency content of a signal drawn so that the energy content in each frequency region and time is displayed on a colored scale. The horizontal axis of the spectrogram is time, and the picture shows how the signal develops and changes over time. The vertical axis of the spectrogram is frequency and it provides an analysis of the signal into different frequency regions. However, this conventional speech representation emphasize many spectro-temporal details that are not directly related to the linguistic information encoded in the speech signal and which consequently do not display the perceptual stability characteristic of human listeners.

## Conclusion

We've concluded that none of the methods exposed above can thoroughly evaluate such aperiodic signal as the erygmophonic speech. Though other methods could be tried especially the speech modeling and the nonlinear dynamic prediction methods that stay at the level of fundamental experimenting field and have not been applied in the clinical practice through established software yet.
